# A New Look at the Chemical Recycling of Polypropylene: Thermal Oxidative Destruction in Aqueous Oxygen-Enriched Medium

**DOI:** 10.3390/polym14040744

**Published:** 2022-02-15

**Authors:** Vadim V. Zefirov, Igor V. Elmanovich, Andrey I. Stakhanov, Alexander A. Pavlov, Svetlana V. Stakhanova, Elena P. Kharitonova, Marat O. Gallyamov

**Affiliations:** 1 A.N. Nesmeyanov Institute of Organoelement Compounds, Russian Academy of Sciences, Vavilova St. 28, 119991 Moscow, Russia; elmanovich@polly.phys.msu.ru (I.V.E.); stakh@ineos.ac.ru (A.I.S.); pavlov@ineos.ac.ru (A.A.P.); glm@spm.phys.msu.ru (M.O.G.); 2 Faculty of Physics, M.V. Lomonosov Moscow State University, Leninskie Gory 1-2, 119991 Moscow, Russia; harit@polly.phys.msu.ru; 3 Department of Analytical Chemistry, Dmitry Mendeleev University of Chemical Technology of Russia, Miusskaya Sq. 9, 125047 Moscow, Russia; sstahanova@muctr.ru

**Keywords:** chemical recycling, plastic waste, polypropylene, polyolefins, thermal destruction, thermolysis, oxidation

## Abstract

Recycling of plastic waste, in particular polypropylene, represents one of the most pressing challenges facing humanity. Despite the promise of chemical methods for recycling polypropylene, they usually require a high temperature and are energy-intensive. In this work, we investigated the oxidative thermolysis of polypropylene in aqueous media. This approach rendered it possible to carry out the decomposition of the polymer at a comparatively low temperature (150 °C). It was shown that among the tested, the most promising aqueous medium for the decomposition of polypropylene is water saturated with gaseous oxygen at an elevated pressure (14 bar) and at a temperature of 150 °C. In such an environment, polypropylene was converted mostly to acetic acid (up to 1.3 g/g acetic acid to starting polypropylene mass ratio). Moreover, methanol, formic acid, and propionic acid were also detected as the products. Finally, the applicability of the proposed recycling method to real polypropylene waste was shown.

## 1. Introduction

Recycling and disposal of plastic waste represents one of the key environmental problems facing humanity [1,2]. Recently, increasing efforts and resources are being spent on new technologies and methods of plastic processing, including at the state level. However, the share of recycled plastic currently accounts for no more than 6% of the total demand for plastics [3]. Packaging materials account for about 40% of the produced plastic, the most common of which are polyethylene (PE) [4], polypropylene (PP) [5], polystyrene (PS) [6] and polyethylene terephthalate (PET) [7].

There are three main strategies for the disposal and recycling of plastic [8,9]: mechanical recycling [10], chemical recycling [11] and the use of plastic waste as fuel [12]. Mechanical recycling implies either direct reuse of uncontaminated discarded plastic into a new product without loss of properties, or crushing and melting of plastic waste into granules (with some loss of molecular weight) [13]. Such recycling requires high-quality purification and sorting of waste, and inevitably leads to deterioration of the properties of the recycled material, which limits the applications of mechanical recycling. Chemical recycling methods render it possible to convert plastic materials into low-molecular-weight compounds, usually liquids or gases, which are suitable for use as raw materials for the production of new petrochemical products and plastics [14,15]. Finally, plastic waste can be used as fuel [16]. Indeed, the incineration of energetic waste materials can create heat or power that can be directly used in technological processes or to heat buildings [17]. This method is commonly used for the treatment of mixed and highly contaminated waste that cannot be easily and (or) economically recycled through any other process.

Among the strategies described, the chemical processing of waste is one of the most ecologically sustainable and economically favorable, since it is highly variable and has significantly less restrictions [18]. One of the promising methods of chemical processing of plastics is thermolysis, i.e., the decomposition of polymers at high temperatures, including the use of catalysts and oxidants [19,20,21]. Depending on the process conditions during thermolysis, a mixture of molecules in the form of a liquid or wax is usually formed as the main product, which can then be processed into certain chemicals or fuel [22,23,24]. Non-catalytic thermolysis of polyolefins requires rather high temperatures (over 500 °C), which means it is energy-intensive. The use of oxygen can significantly reduce the temperature of polymer degradation and change the composition of the resulting products [25,26].

Another known method that allows one to lower the decomposition temperature of polymers and narrow the range of products obtained is the use of supercritical (sc) fluids, mainly water or alcohols [27,28]. It is important to note that the sc fluid-assisted decomposition strategy is usually used for condensation polymers with ester or amide bonds, primarily polyethylene terephthalate and polyamides. A relatively small number of works are devoted to the decomposition of PE and PP in sc fluids (specifically in water); however, these works have shown that the sc medium makes it possible to increase the efficiency of the thermal degradation process [29,30,31]. In this case, sc water acts as a solvent, which helps to fix the issues of poor heat transfer and high viscosity of the medium. In addition, sc water at temperatures of 500–800 °C also acts as a hydrogen donor, which promotes cracking and gasification of plastic [32]. However, a rather high temperature and pressure are required to achieve the sc state for water (critical temperatures and pressures are 373 °C and 220 bar, respectively).

Among a wide range of other sc fluids, supercritical carbon dioxide (sc CO_2_) is the most convenient for possible industrial applications (critical temperatures and pressures are 31.1 °C and 70 bar, respectively). This cheap, non-toxic solvent has discovered its way into a wide variety of processes, but is rarely used in plastic degradation processes. In the literature, the use of sc CO_2_ in such processes is described only either as a medium for sc extraction of various contaminants from plastics [33,34] or as a plasticizer in the process of catalytic hydrolysis of PET [35,36] and oxidative thermolysis of nylon [37]. To the best of our knowledge, the only work to date, in which the thermal decomposition of polyolefins is carried out directly in an sc CO_2_ medium, discloses the concept of thermal oxidation of polypropylene in an sc CO_2_ medium, which rendered it possible to completely decompose the polymer in the presence of a catalyst at relatively low temperatures (135 °C) [38].

Moreover, it is known that polyolefins are susceptible to oxidation in peroxycarbonic acid solutions. This acid is formed upon saturation of an aqueous solution of hydrogen peroxide with carbon dioxide under high pressures: H_2_O_2_ + CO_2_ → HOCOOOH [39]. Earlier, our research group demonstrated the possibility of functionalization of a polyolefin membrane in peroxycarbonic acid at room temperature [40,41]. Finally, it is known that polyolefins are susceptible to oxidative degradation when exposed to hot water or steam [42,43]. All these facts suggest that in polar aqueous media in the presence of oxygen, the oxidative thermolysis of polypropylene should proceed quite efficiently. Therefore, the aim of the work was to study various oxygen-enriched aqueous media (including in the presence of sc CO_2_) for the decomposition of polypropylene. Unlike our previous work, we did not use additional catalytic materials here [38]. At the same time, in contrast to the conditions of decomposition in sc water, the range of temperatures and pressures proposed in this work are much lower. This means that the proposed method from a technological point of view is much more acceptable for use in real processes. The following thermolysis media were studied and compared in the work: H_2_O_2_; H_2_O_2_ + sc CO_2_; H_2_O + O_2_ and H_2_O + O_2_ + sc CO_2_. The polymer destruction products were studied by nuclear magnetic resonance spectroscopy (NMR), gas chromatography–mass spectrometry (GC–MS), thermogravimetric analysis (TGA), and Fourier transform infrared spectroscopy (FT-IR) methods.

## 2. Materials and Methods

Polypropylene was provided by NKT company (Denmark) in the form of round granules, the same batch of polypropylene granules was used in our previous work (homopolymer, crystallinity ~40%, isotacticity > 90%, melting point 158 °C, polymer fusion enthalpy 62 J g^−1^, molar mass unspecified) [38]. Aqueous hydrogen peroxide solution (≥35%, Aldrich) was used to obtain 30% H_2_O_2_ aqueous solution. Milli-Q water (degree of purification) was obtained using a Milli-Q Integral System (Millipore S. A. S., Molsheim, France). For all high-pressure experiments, a homemade stainless-steel high-pressure autoclave with a titanium jacket and a PTFE seal was used.

**Figure 1:**
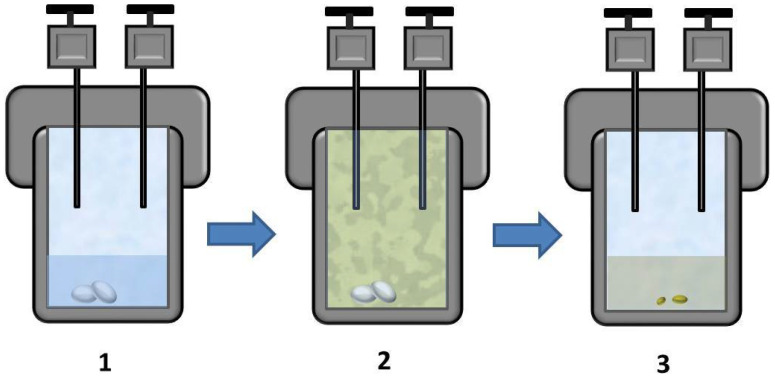
Schematic representation of experiments. Step 1: a weighed portion of the polymer is placed in 1 mL of the liquid phase in a pressure autoclave. Step 2: oxygen and/or carbon dioxide is injected (O_2_ can also be produced due to the decomposition of H_2_O_2_) into the autoclave and heated, thermal oxidation and decomposition of the polymer begins. Step 3: Liquid reaction products and residual solids are retrieved from the autoclave.

The thermal destruction of PP was carried out as follows. A polymer sample was placed in a high-pressure 23 mL autoclave together with a given volume (1 mL) of the liquid polar phase (30% aqueous hydrogen peroxide solution or Milli-Q water). This amount of water provided a pressure of 4.7 bar at 150 °C (according to the NIST Chemistry WebBook program, National Institute of Standards and Technology, Gaithersburg, MD, USA). Taking into account the decomposition of hydrogen peroxide into water and gas gives a pressure change for water vapor of less than 0.1 bar, therefore for both media (H_2_O + O_2_ and H_2_O_2_), the water vapor pressure is the same. In addition, it is important to note that the stainless-steel walls of the autoclave clearly act as a catalyst for the decomposition of hydrogen peroxide, but this effect was not studied in detail in this work. The autoclave was sealed and then for some samples oxygen and/or carbon dioxide was injected therein. The detailed parameters for each experiment are presented in Table 1. To avoid generating an excessively high pressure of carbon dioxide, the autoclave was preheated to 40 °C and pressurized to 90 bar, which corresponded to a carbon dioxide density of 0.51 g/mL (according to the NIST Chemistry WebBook program, National Institute of Standards and Technology, USA) and in turn corresponded to 313 bar at 150 °C. The mass of free oxygen (excluding oxygen atoms contained in water) in each experiment was 200 mg (corresponding to elevated pressure of ca. 7.1 bar at 40 °C and ca. 9.6 bar at 150 °C). The pressure in the autoclave in each experiment was the sum of the partial pressures of water vapor, oxygen, and carbon dioxide (if present). The weighed portions of PP were 60 mg. Thus, the free oxygen/polymer mass ratio in all experiments was 3.3. After filling the autoclave, it was placed in a thermostat (Binder, Germany) at a temperature of 150 °C for 24 h. This temperature was chosen based on our preliminary experiments. We have shown that at temperatures below 130 °C, much less complete decomposition of the polymer occurs (most of it remains in the solid phase). On the other hand, we could not select a higher temperature for the experiments due to some technical limitations: at temperatures above 150 °C, the polymer seal of a high-pressure autoclave can soften and break the tightness of the autoclave. After 24 h of exposure, the autoclave was slowly decompressed and opened, and the resulting material was retrieved. In addition, for comparison, the thermal decomposition of PP was carried out at the same temperature in water without the addition of oxygen (sample PP_0). Figure 1 schematically depicts the experiments.

Pristine PP and liquid products of thermal decomposition of PP were analyzed by means of thermogravimetric analysis (using a NETZSCH STA 449 C device, Selb, Germany). Liquid products with solid residues were first stirred vigorously, and then samples were obtained with a pipette and placed inside Al crucibles. The samples were heated from 30 to 590 °C at a rate of 5 °C min^−1^ in an air atmosphere.

To obtain transmission IR spectra, an FT-IR spectrometer Nexus (Thermo Fisher scientific, Waltham, MA, USA) was used. The obtained data was used to detect changes in the polymer structure after oxidative thermolysis. To obtain a solid phase, all liquid samples were dried at 100 °C. By preparing compressed KBr tablets, the solid phase was analyzed. Transmission spectra were recorded from 650 to 4000 cm^−1^. Each spectrum is an average of 32 scans with a resolution of 1 cm^−1^.

^1^H, ^13^C{^1^H} and ^1^H-^13^C HMQC NMR spectra were obtained with a Bruker Avance 600 FT-NMR spectrometer (^1^H frequency 600.22 MHz). For sample preparation, 50 µL of D_2_O was added to liquid products with no prior purification or filtration of the products. The measurements were performed using the residual signals of this deuterated solvent (^1^H at 4.7 ppm). ^13^C NMR chemical shifts were referenced to external TMS. The water residual signal in ^1^H spectra were suppressed using a Watergate W5 pulse sequence [44]. Gas-chromatography–mass spectroscopy was performed on a Focus DSQ GC (Thermo Fisher scientific, USA) device with electron impact ionization, equipped with a TR-WAX-30 m polar column (Thermo Fisher scientific, USA), 30 m in length. Before samples were taken, the products were centrifuged. The quantitative determination of molar concentration of acids was carried out by potentiometric titration on an automatic titrator ATP-02 (Akvilon, Podolsk, Russia) using a 0.1 M NaOH solution as a titrant.

## 3. Results

### 3.1. Characterization of Obta ined Products

**Figure 2:**
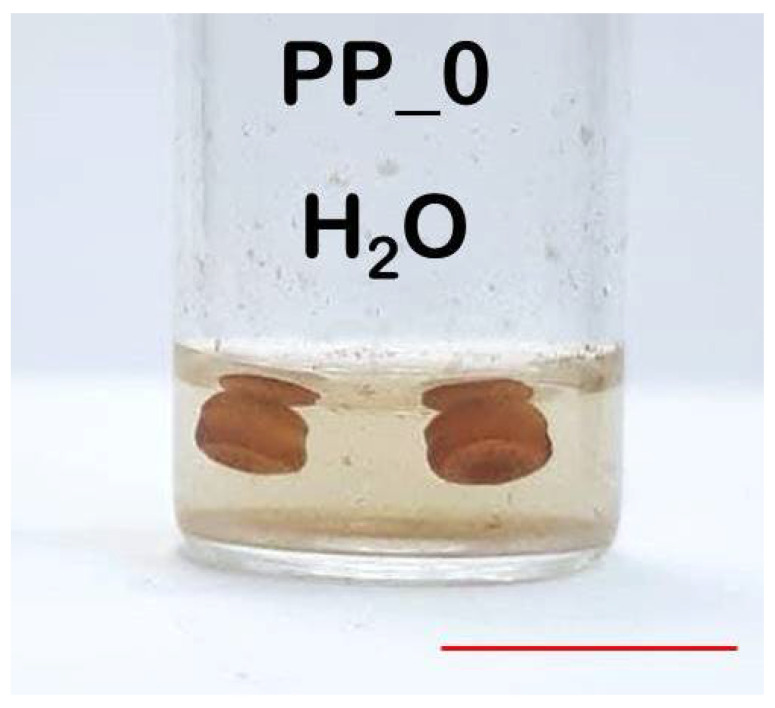
Photograph of the PP_0 sample obtained by thermolysis of PP in a sealed autoclave with water at 150 °C. The scale line is 1 cm.

After the thermolysis procedure, all samples obtained were significantly different visually. Nevertheless, in all samples obtained in oxygen enriched media (PP_1, PP_2, PP_3 and PP_4), complete or significant decomposition of the PP phase was observed. At the same time, in a pure aqueous medium (sample PP_0), there was no significant decomposition of PP granules after hydrothermal treatment at 150 °C (Figure 2). The saturated water vapor pressure at this temperature was 4.7 bar. One can observe that the initially transparent water became colored. ^1^H NMR spectrum of the liquid product shows peaks that correspond to acetic acid and acetone with traces of formic acid and methanol, but the signals are almost negligible as compared to the water peak (Figure S1). Further, it can be seen that the granules oxidized (darkened), but retained the sizes close to the original ones. This oxidation appears to be due to the small amount of residual oxygen from the air in the autoclave. The mass loss of the solid phase of the products, measured gravimetrically, was no more than 2%. This experiment suggests that these conditions were insufficient for the effective decomposition of PP, which was also confirmed by the obtained TGA and NMR data.

**Figure 3:**
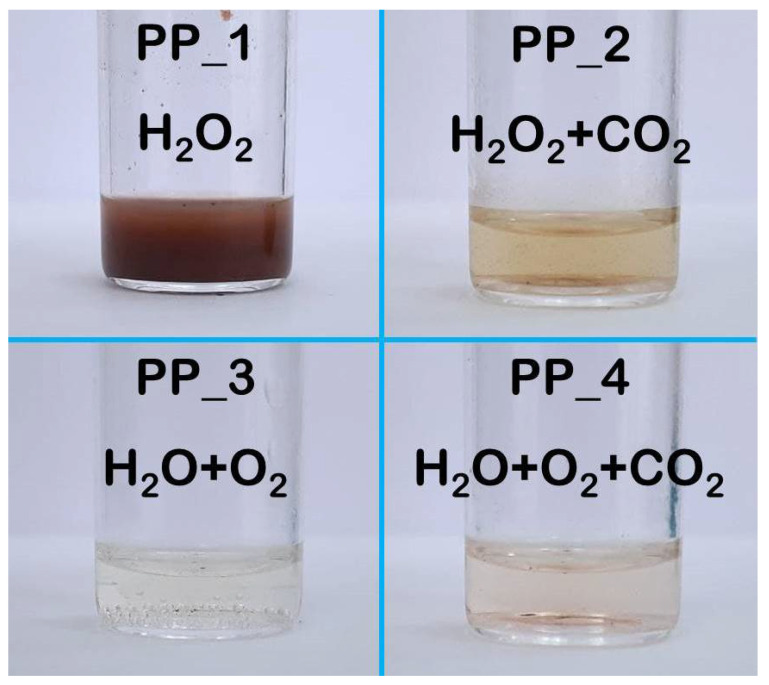
Photograph of samples, obtained by thermolysis of PP at 150 °C in a sealed autoclave with aqueous media in the presence of oxygen at elevated pressure and (right column) additionally sc CO_2_ at high pressure.

After thermolysis of PP in the presence of oxygen in the autoclave at 150 °C and elevated pressure, the obtained samples appeared significantly different (Figure 3). The main difference was the absence of PP granules, which either disappeared completely or remained in the form of small dispersed particles. Moreover, it can be seen that thermolysis was the least efficient in the autoclave with H_2_O_2_ medium. A turbid brown liquid with small oxidized PP particles interspersed was observed. However, even for the decomposition in H_2_O_2_ as compared to water, the ^1^H NMR spectra of the liquid products with the water peak not suppressed demonstrates a significant increase in the product signal intensities (see Figure S1). At the same time, the addition of sc CO_2_ at a high pressure to the autoclave with hydrogen peroxide made it possible to change significantly (at least by appearance) the products obtained. Thus, in PP_2 sample, there were no more noticeable particles of oxidized PP, and the liquid phase became transparent and slightly colored. This result could be expected, since we have previously demonstrated a positive effect of sc CO_2_ on the efficiency of PP oxidative thermolysis [38]. Surprisingly, the most homogeneous, uncolored and transparent liquid medium was obtained for the PP_3 sample obtained by thermolysis in the autoclave with water and oxygen at an elevated pressure. When sc CO_2_ at high pressure was added to the autoclave with this decomposition medium (H_2_O + O_2_), the PP_4 sample was obtained, which also had high transparency and only slightly differed in color. Thus, already at the stage of visual analysis of the results obtained, it was possible to draw a conclusion about the effectiveness of the oxidative pyrolysis in the aqueous media.

**Figure 4:**
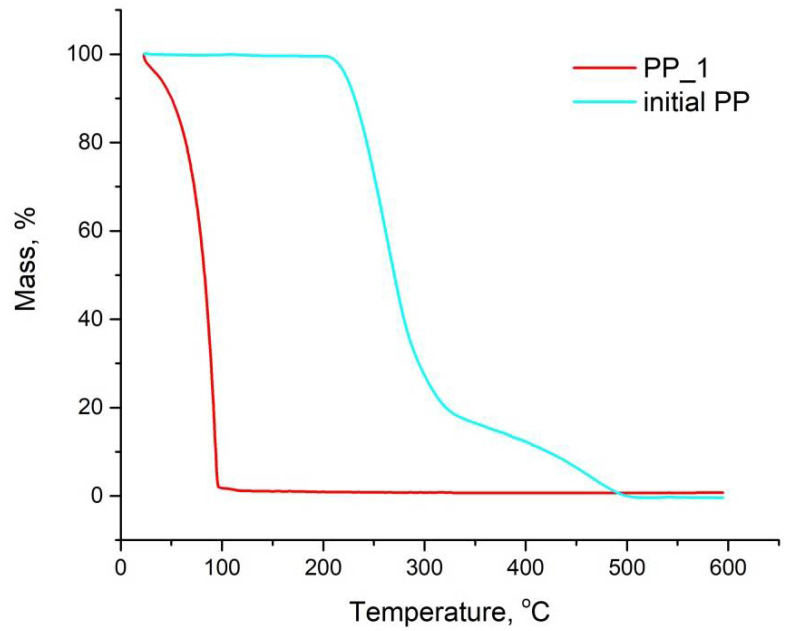
TGA data for sample PP_1, obtained by oxidative thermolysis at 150 °C in H_2_O_2_, and initial solid PP, used in all experiments.

Figure 4 shows the TGA data for the PP_1 sample and the initial solid PP. It is clearly seen that the absolute majority of mass of the PP_1 sample was water and volatile components. The percentage of non-volatile phase in this sample was less than 1.5% of the total sample weight, which is equivalent to 7% of the initial weight of PP in the experiment. The TGA data for all samples appear the same, differing only in the amount of the non-volatile phase. For sample PP_1 obtained in medium H_2_O_2_, this amount is approximately 2 times higher than for other samples. However, these data are at the limit of the instruments accuracy, so they should be taken as estimates.

**Figure 5:**
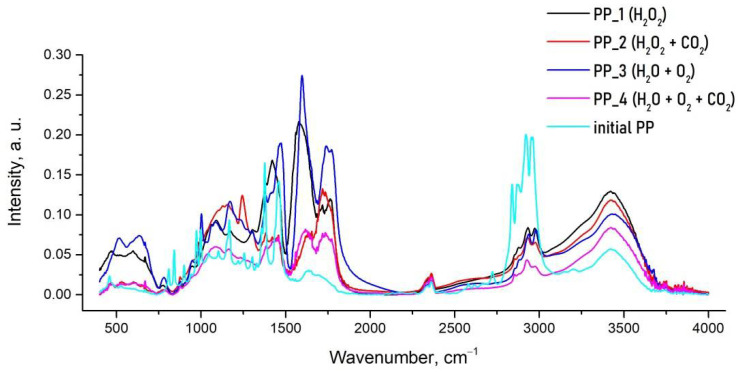
Typical FTIR spectra for the initial PP and for the solid products of thermal oxidation of PP at 150 °C in a sealed autoclave with aqueous media in the presence of oxygen.

Using FTIR spectroscopy, we analyzed the solid phase remaining in all samples (Figure 5). In the IR spectra of solid products of PP oxidation, the following changes are observed in comparison with the spectrum of the initial PP. The vibrations peaks in the region 2850–2960 cm^−1^ are related to the stretching mode of the υ(C–H) bond in the –CH_3_ and –CH_2_ groups [45]. The intensity of these bands in the spectra of the oxidation products decreases, but does not disappear completely, which indicates a decrease in the number of methyl and methylene groups in the structure of the decomposition products in comparison with the initial polymer. This effect is especially pronounced for the PP_4 sample, the oxidation of which was carried out in H_2_O + O_2_ medium in the presence of sc CO_2_. An important indicator of the degree of destruction of the PP polymer chain is a decrease in the intensity of the 2960 cm^−1^ band as compared to the 2920 cm^−1^ band, which correspond to the stretching vibrations of CH at the tertiary and secondary carbon atoms, respectively. The appearance of this effect also indicates a deeper destruction of the polymer chain for the oxidation product PP_4 in comparison with the products PP_1, PP_2 and PP_3.

In the spectra of all degradation solid products, there are no bands in the range of 800–1000 cm^–1^, which are present in the initial PP and are due to the conformational regularity of the polymer chain, i.e., isotactic structure of the initial PP. This fact also indicates a deep destruction of the polymer chain of the oxidation products, leading to the disappearance of the ordered supramolecular structure of the polymer.

In turn, the appearance of oxygen-containing oxidation products of a complex structure is observed, as evidenced by the appearance of a wide band in the region of 3350–3500 cm^–1^, which corresponds to the stretching vibrations of the hydroxyl group [46]. A wide band in the region of 1050–1150 cm^−1^ may also indicate the presence of a hydroperoxide group –OOH in the products. The appearance of intense bands in the 1720–1740 cm^−1^ region indicates the presence of C=O groups, and in the 1440–1460 cm^−1^ region the presence of C–O bonds manifests itself.

It should be emphasized that the solid products of PP_1 and PP_3 samples obtained in the absence of sc CO_2_ are characterized by the appearance of intense bands in the range of 1580–1600 cm^−1^, which apparently correspond to the presence of a significant amount of carboxyl groups in the composition of the oxidation products. Thus, the solid products of PP oxidation are mixtures of oligomeric products containing a large amount of oxygen-containing functional groups of various nature. The products of oxidation carried out in a sc CO_2_ medium are distinguished by a deeper degree of destruction, which is especially pronounced for PP_4 sample.

### 3.2. Chemical Composition of Liquid Products

**Figure 6:**
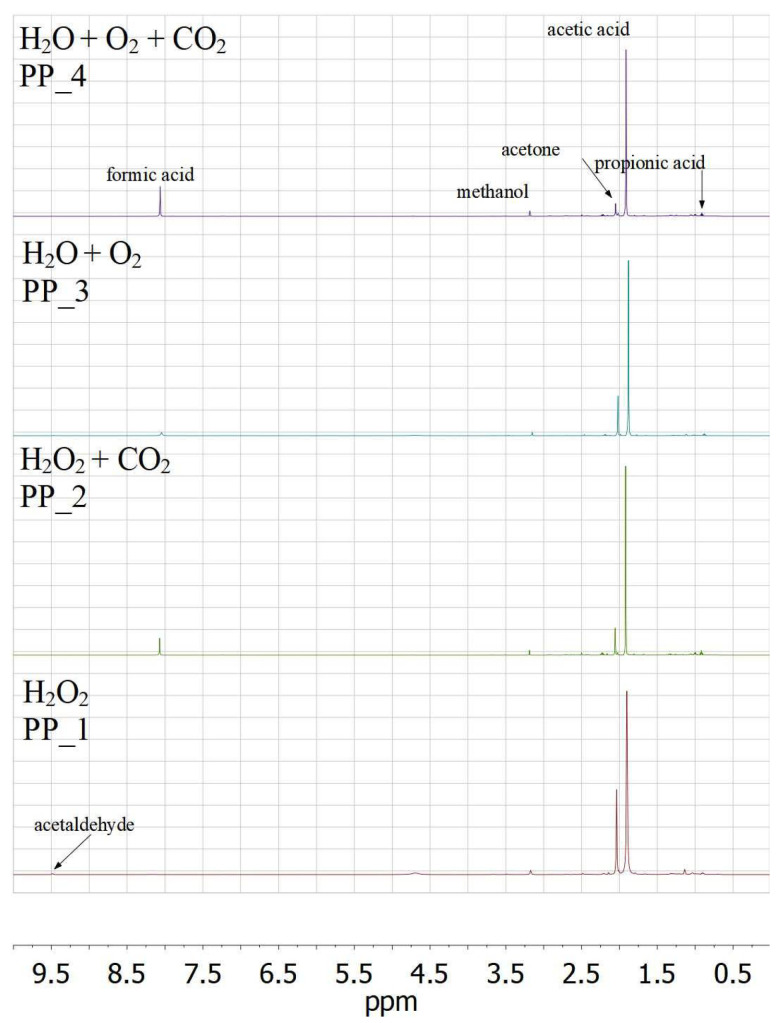
^1^H NMR spectra with suppressed water signal of thermal oxidation products obtained at 150 °C in a sealed autoclave with various media.

Samples for NMR analysis were obtained from the original aqueous solutions of the products with some amount of D_2_O. ^1^H NMR spectra of the liquid products of PP decomposition in various media with a suppressed water peak are presented in Figure 6. One can see that each spectrum has the most prominent peak at around 1.92 ppm, and a neighboring signal at 2.06 ppm. In order to definitely assign these neighboring signals in ^1^H spectrum, ^1^H-^13^C HMQC NMR spectrum for PP_1 sample was obtained (Figure S3). This spectrum clearly shows correlations between CH_3_-protons of acetone and acetic acid with the corresponding carbon nuclei. These correlations allow assignment of the proton signal at 1.92 ppm to acetic acid and the signal at 2.06 ppm to acetone. The other identified compounds are formic acid (8.05 ppm), methanol (3.19 ppm), propionic acid (0.93 ppm) and acetaldehyde (9.5 ppm). For all the detected products, ^13^C NMR were obtained (see Figure S2). Signals of acetone, methanol and propionic acid are present in each ^1^H NMR spectra. The formic acid peak is much less pronounced for the H_2_O_2_ medium than for all other media. Acetaldehyde is detected only for the decomposition in H_2_O_2_ medium. The presence of acetic acid, formic acid and propionic acid was also confirmed by means of GC–MS (see Figures S5–S8).

Table 2 presents molar distribution of acids, normalized to the sum of the content of all acids, as revealed by ^1^H NMR and GC–MS methods. One can see that the data obtained by the two methods are in general agreement. For all samples, the molar content of acetic acid is the highest one, while for propionic acid only traces with no more than 9 mol. % are detected. According to ^1^H NMR data, for the decomposition in H_2_O_2_ medium, the presence of sc CO_2_ leads to formation of larger amount of formic acid. However, it is not confirmed by GC–MS method, which gives the similar molar percentage of formic acid for H_2_O_2_ and H_2_O_2_ + CO_2_ media. The case of the decomposition in H_2_O_2_ is the only one in which there is a discrepancy between ^1^H NMR and GC–MS data: the data of the two methods is in good agreement for all other samples. Indeed, for the H_2_O_2_ medium, ^1^H NMR detects only traces of formic acid, while GC–MS detects 26 mol. % formic acid. For the decomposition in H_2_O + O_2_ medium, the molar content of acetic acid is slightly higher than that for the decomposition in H_2_O + O_2_ + CO_2_. 

In order to quantitatively assess the efficiency of trecycling of PP into low-molecular-weight products, potentiometric titration was performed and the molar concentration of acids in the products was measured. Further, the measured concentrations were used to estimate the overall mass of the acids in the products from the known values of mass ratio of the sample to the total product and from the acetic acid/formic acid/propionic acid molar ratios calculated from ^1^H NMR. The corresponding values for the molar concentrations of acids and the masses of acetic acid, formic acid and propionic acid are presented in Table 3. Before discussing the obtained values, we should address the errors for the values given in Table 3. Potentiometric titration gives an accurate molar concentration with no more that 1% relative error. However, to determine the total mass content, it is necessary to take into account several sources of errors, the main of which are error from ^1^H NMR peak integrations, which was performed to calculate the molar ratios of the acids, and the error derived from calculating total molar acid content in the product from the molar acid concentration obtained via potentiometric titration. According to our estimates, the total relative error for the acid content values does not exceed 25%. For the products of thermal decomposition performed in H_2_O_2_ medium, a clear influence of the sc CO_2_ presence on the yield of the acids is observed. Indeed, the molar concentration of acids is equal to 0.6 mol/L for the decomposition in H_2_O_2_ and reaches 1.0 mol/L for the decomposition in H_2_O_2_ + CO_2_. The corresponding masses of the sum of all acids produced in H_2_O_2_ and H_2_O_2_ + CO_2_ are calculated to be 30 mg and 60 mg, respectively. For the decomposition in H_2_O + O_2_ based media, the influence of sc CO_2_ presence is less evident. Indeed, both the molar acid concentration and the total acid content values are close for the H_2_O + O_2_ and H_2_O + O_2_ + CO_2_ samples. For the decomposition without sc CO_2_ presence, the values are slightly higher and the total acid content reaches 80 mg, which is more than the mass of pristine PP before decomposition (60 mg). The presented values are in general agreement with the values, derived from ^1^H NMR spectra recorded without water suppression (the corresponding spectra are presented in Figure S4). ^1^H NMR gives the value of 50 mg as a maximum total acid content for the PP_3 sample, confirming that decomposition in H_2_O + O_2_ medium gives the highest product yield. However, the ^1^H NMR data seems to be less precise due to the pronounced broadening of the ^1^H signal of water.

### 3.3. Chemical Decomposition of Real Plastic Waste

**Figure 7:**
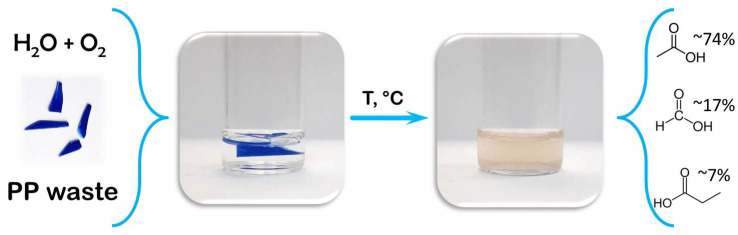
Schematic illustration of an experiment on the chemical processing of real PP waste.

To study the applicability of the described approach to the chemical processing of real PP waste, we conducted an experiment on the decomposition of PP packaging from ice cream (Figure 7). H_2_O + O_2_ was chosen as the decomposition medium, firstly, as the simplest technically, and secondly, as the most effective in the conversion of PP into acids. For the experiment, 60 mg of the ice cream packaging were obtained. All other conditions were as described above. In line with our expectations, the decomposition products of real PP waste were in agreement with the data obtained in model experiments with pure PP (Figure S9). The main decomposition product was acetic acid; while methanol, formic acid, and propionic acid were also detected.

## 4. Discussion

This work was the first to demonstrate the oxidative thermolysis of PP in aqueous media at relatively low (150 °C) temperatures and elevated or high pressures. The data obtained show that for the same amount of free (not included in water molecules) oxygen, the process of PP decomposition in H_2_O + O_2_ and H_2_O_2_ media produces different results. We assume that this phenomenon may be related to the kinetics of the decomposition of hydrogen peroxide. This is an unexpected result, which requires careful further research. Moreover, in accordance with expectations, the influence of sc CO_2_ on the process of PP decomposition was shown. It is known that, like many polymers, PP swells in sc CO_2_ [47]. Therefore, the addition of sc CO_2_ to the autoclave used for PP decomposition leads to an enhanced transport phenomena in the polymer bulk. Accelerated mass transfer and faster oxidation lead to a change in the kinetics of the decomposition process and a variation in the balance of the reaction products, which is clearly seen in the example of samples PP_1 and PP_2. With the addition of sc CO_2_, the amount of acids obtained doubles (30 mg for PP_1 and 60 mg for PP_2). In addition, their composition changes, with more formic acid present in the case of decomposition in H_2_O_2_ + CO_2_. At the same time, the addition of sc CO_2_ to the H_2_O + O_2_ medium led to a slight decrease in the amount of acids, while their composition did not change significantly. The slight decrease in the H^+^ concentration and the calculated acid content for the decomposition in H_2_O + O_2_ + CO_2_ as opposed to the decomposition in H_2_O + O_2_ is probably due to the partial extraction of the decomposition products during the decompression procedure. Thus, we observe that sc CO_2_ enhances the thermal decomposition of PP in the case of a less reactive medium, i.e., H_2_O_2_. A similar result was previously obtained for the PP decomposition in pure oxygen, where addition of sc CO_2_ accelerated the decomposition process in the case of a low oxygen content for non-catalytic thermal oxidation and did not affect significantly catalytic decomposition at an optimal O_2_:PP ratio [38]. The observed enhancement is probably related to the aforementioned ability of sc CO_2_ to induce polymer swelling as well as to act as a solvent for the low-molecular-weight destruction products, accelerating the decomposition process. It was also shown that the reaction products formed during oxidative thermolysis of PP in polar aqueous media generally correspond to the products obtained during oxidative thermolysis in pure oxygen [38]. Acetic acid was the main product; while formic acid, propionic acid, acetone, and methanol were found in smaller quantities.

The maximum calculated acid content was 1.3 g/g to the starting polypropylene mass for the polymer decomposition in H_2_O + O_2_ medium. Thus, the mass of the acids formed is higher than the mass of the original polypropylene (the additional mass is due to the oxygen involved in the acetic acid formation). Note that the maximum theoretically possible acid content in that case (if all the carbon atoms of the decomposed polymer participate only in the formation of acetic acid, formic acid and propionic acid at calculated molar percentages) is about 2.7 g/g (acid/polypropylene). Thus, the estimated efficiency of the low-molecular-weight product formation in this case is around 50% (in fact, it should be slightly higher, taking into account that acetone and methanol, which do not contribute to molar acid concentration, are also among the low-molecular-weight products). In addition, it should be noted that part of the polymer could turn into a gas fraction, the analysis of which is beyond the scope of this work. Moreover, it should be emphasized that the presented work was performed on a small laboratory scale and concerns pristine polypropylene, which renders the estimation of the process efficiency a preliminary one. When applied to polymer waste, the factors that are unaccounted for in the present work may play an important role, such as the presence of impurities and additives in the feedstock, as well as other possible scaling up issues. However, taking these comments into account and recognizing that there are certainly opportunities for the process optimization, we believe that the results obtained suggest that the presented approach to the chemical recycling of polypropylene is promising. In addition, we confirmed that the chemical processing of real PP waste produces a similar composition of the products, which may indicate the high prospects of the proposed method in real applications.

## Figures and Tables

**Table 1:** Experimental parameters. Free oxygen is the oxygen that is not included in water molecules.

Decomposition Medium	Sample	Polymer Mass, mg	Free Oxygen Mass, mg	CO_2_ Density, g/mL	Total Pressure at 150 °C, Bar
H_2_O	PP_0	60	0	0	4.7
H_2_O_2_	PP_1	60	200	0	14.3
H_2_O_2_ + CO_2_	PP_2	60	200	0.51	327.6
H_2_O + O_2_	PP_3	60	200	0	14.3
H_2_O + O_2_ + CO_2_	PP_4	60	200	0.51	327.6

**Table 2:** Molar distribution of acids, normalized to total acid content, for the products obtained in a sealed autoclave with various oxidative media, according to ^1^H NMR and GC–MS data.

Medium	Acetic Acid, mol. %		Formic Acid, mol. %		Propionic Acid, mol. %	
	^1^H NMR	GC-MS	^1^H NMR	GC-MS	^1^H NMR	GC-MS
H_2_O_2_	96	71	3	26	1	3
H_2_O_2_ + CO_2_	69	70	27	21	3	9
H_2_O + O_2_	81	74	17	19	2	7
H_2_O + O_2_ + CO_2_	60	73	37	21	3	6

**Table 3:** Molar concentration of acids measured by potentiometric titration and total acid content, calculated for the products of polypropylene decomposition in a sealed autoclave with various media.

Sample/Medium	Molar Concentration of Acids, mol/L	Calculated Total Acid Content, mg
PP_1/H_2_O_2_	0.6	30
PP_2/H_2_O_2_ + CO_2_	1.0	60
PP_3/H_2_O + O_2_	1.1	80
PP_4/H_2_O + O_2_ + CO_2_	0.9	60

## Data Availability

Data is contained within the article or Supplementary Material The data presented in this study are available in Supplementary.

## Supplementary Materials

The following are available online at https://www.mdpi.com/article/10.3390/polym14040744/s1, Figure S1: ^1^H NMR spectra of the products of thermal oxidation of PP in a sealed autoclave with water and with hydrogen peroxide at 150 °C; Figure S2: ^13^C NMR spectra of thermal oxidation products obtained in a sealed autoclave with various oxidative media at 150 °C; Figure S3: ^1^H-^13^C HMQC NMR spectrum, obtained for the products of thermal decomposition in a sealed autoclave with H_2_O_2_ at 150 °C (PP_1); Figure S4: ^1^H NMR spectra without water suppression of thermal oxidation products obtained in a sealed autoclave with various oxidative media at 150 °C; Figure S5: GC–MS spectrum for the products of thermal destruction of polypropylene in a sealed autoclave with H_2_O_2_ at 150 °C; Figure S6: GC–MS spectrum for the products of thermal destruction of polypropylene in a sealed autoclave with H_2_O_2_ + CO_2_ at 150 °C; Figure S7: GC–MS spectrum for the products of thermal destruction of polypropylene in a sealed autoclave with H_2_O + O_2_ at 150 °C; Figure S8: GC–MS spectrum for the products of thermal destruction of polypropylene in a sealed autoclave with H_2_O + O_2_ + CO_2_ at 150 °C; Figure S9: ^1^H NMR spectra of the product of decomposition of plastic waste. Lower image without water suppression, upper image with water suppression.
