# Supplementary material for: A New Look at the Chemical Recycling of Polypropylene: Thermal Oxidative Destruction in Aqueous Oxygen-Enriched Medium
Source: Polymers (Basel). 2022 Feb 15;14(4):744. doi: 10.3390/polym14040744 (PMC8878291; doi:10.3390/polym14040744)
Supplement: Supplementary file 1 [file polymers-14-00744-s001.zip › polymers-1511808-supplementary.pdf]

A new look at the chemical recycling of polypropylene: thermal oxidative destruction in aqueous oxygen-enriched medium

Vadim V. Zefirov [1,2,*], Igor V. Elmanovich [1,2], Andrey I. Stakhanov [1], Alexander A. Pavlov [1], Svetlana V. Stakhanova [3], Elena P. Kharitonova [2] and Marat O. Gallyamov [1,2]

Supplementary information

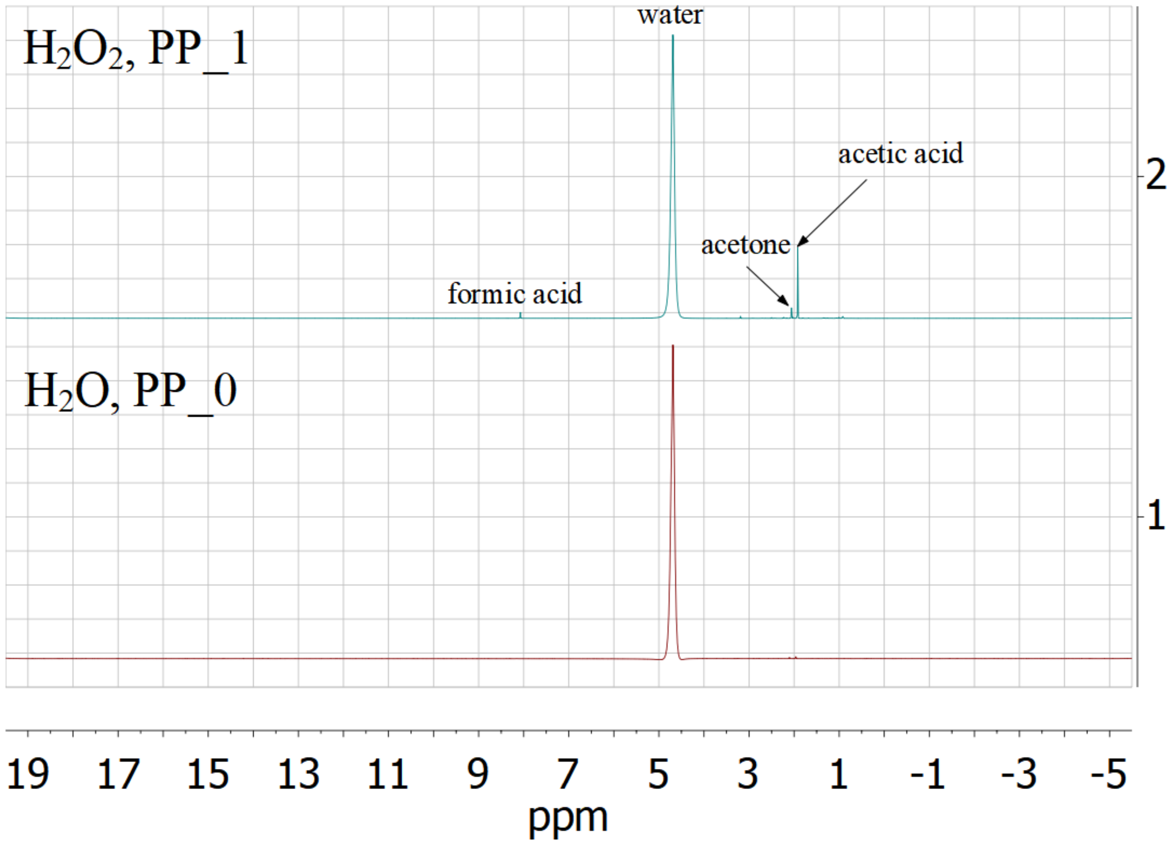

Figure S1. ^{1}H NMR spectra of the products of thermal oxidation of PP in a sealed autoclave with water and with hydrogen peroxide at 150 °C.

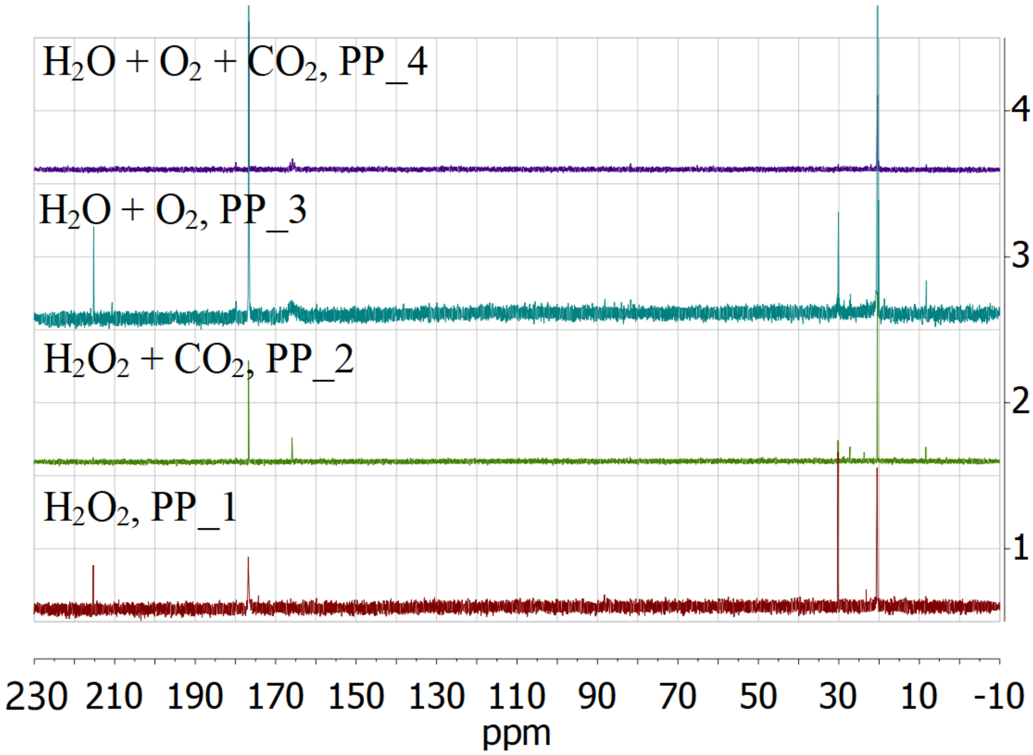

Figure S2. ^{13}C NMR spectra of thermal oxidation products obtained in a sealed autoclave with various oxidative media at 150 °C.

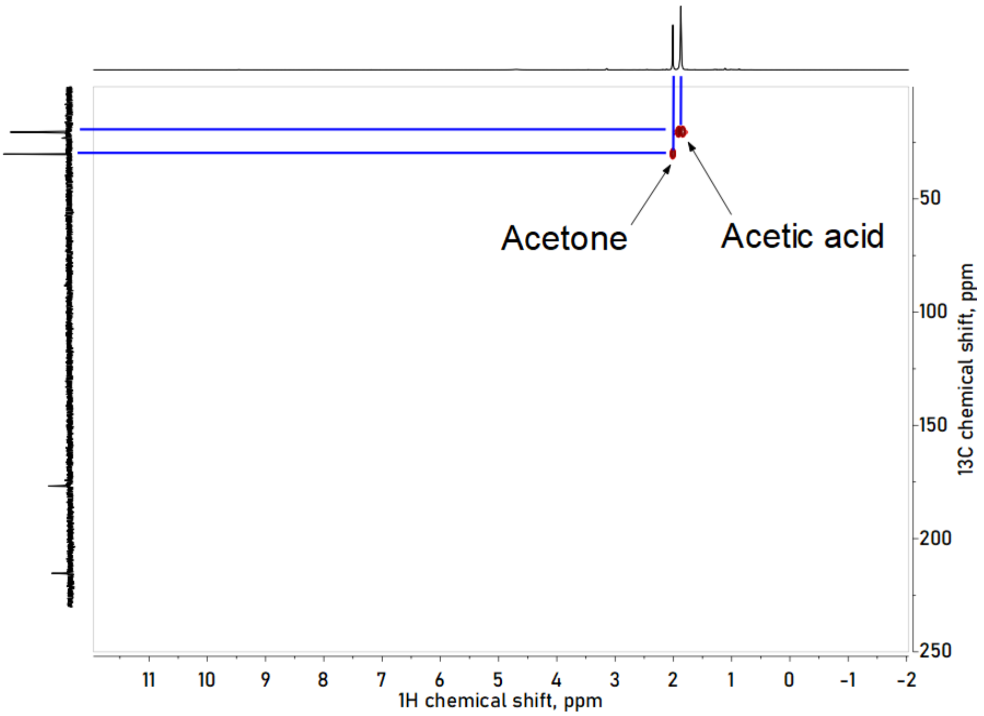

Figure S3. ^{1}H-^{13}C HMQC NMR spectrum, obtained for the products of thermal decomposition in a sealed autoclave with H_2O_2 at 150 °C (PP_1).

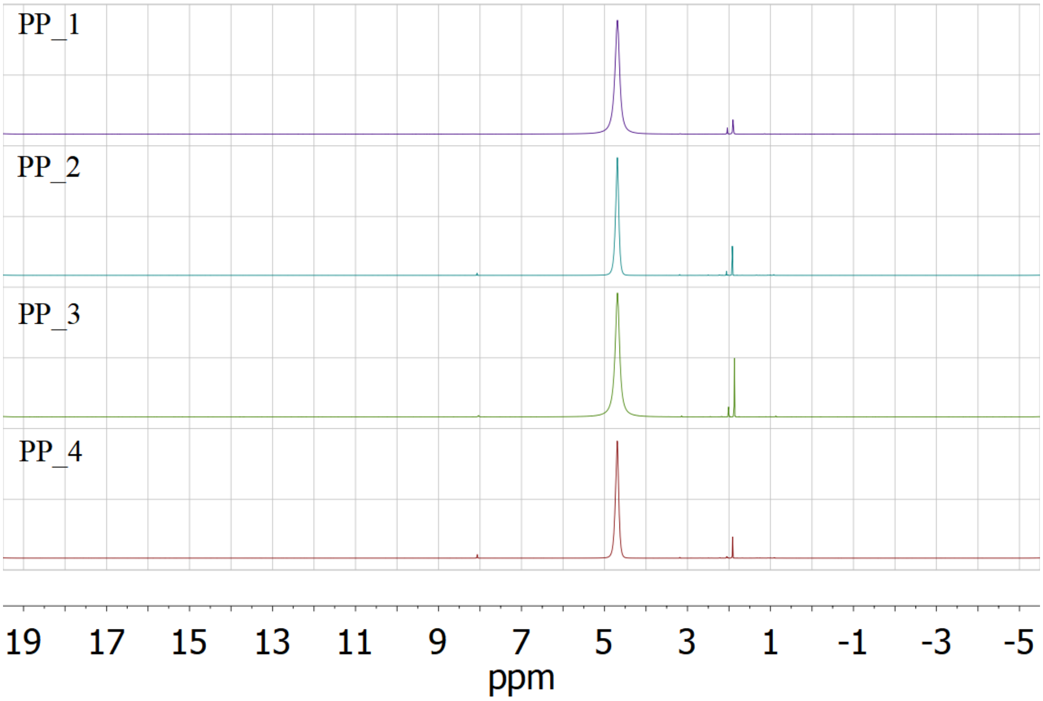

Figure S4. ^{1}H NMR spectra without water suppression of thermal oxidation products obtained in a sealed autoclave with various oxidative media at 150 °C.

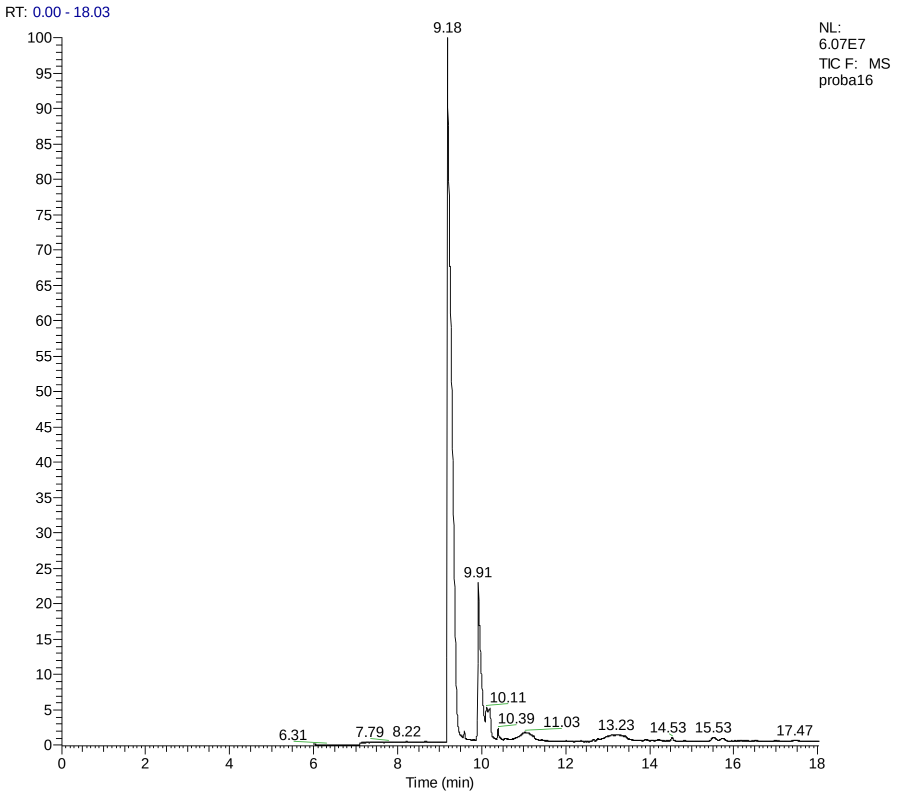

Figure S5. GC-MS spectrum for the products of thermal destruction of polypropylene in a sealed autoclave with H_2O_2 at 150 °C.

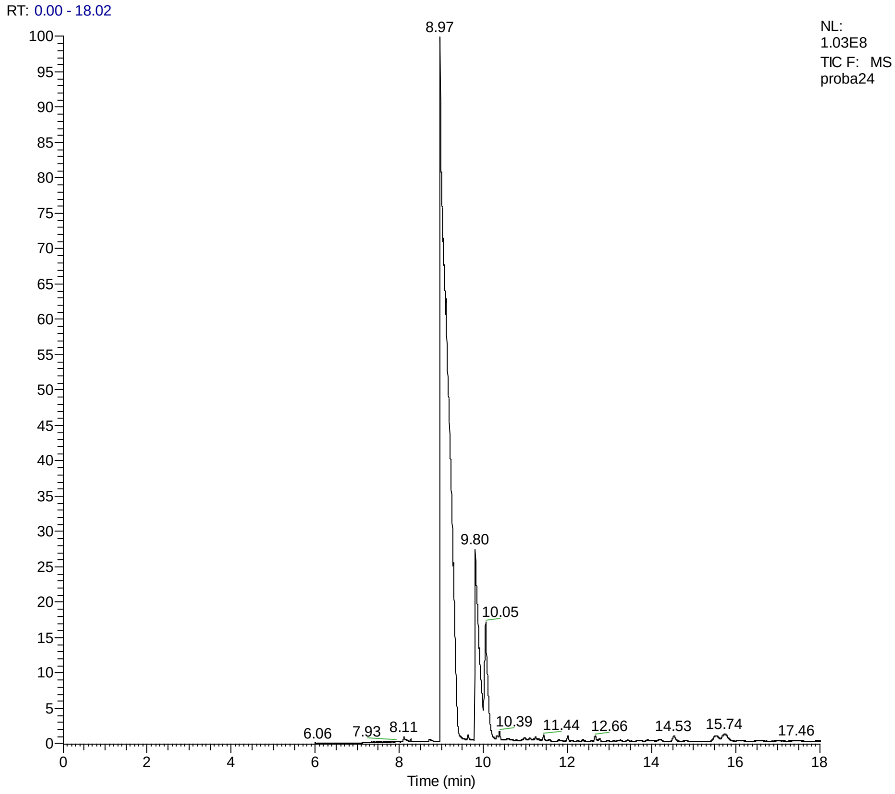

Figure S6. GC-MS spectrum for the products of thermal destruction of polypropylene in a sealed autoclave with H_2O_2 + CO_2 at 150 °C.

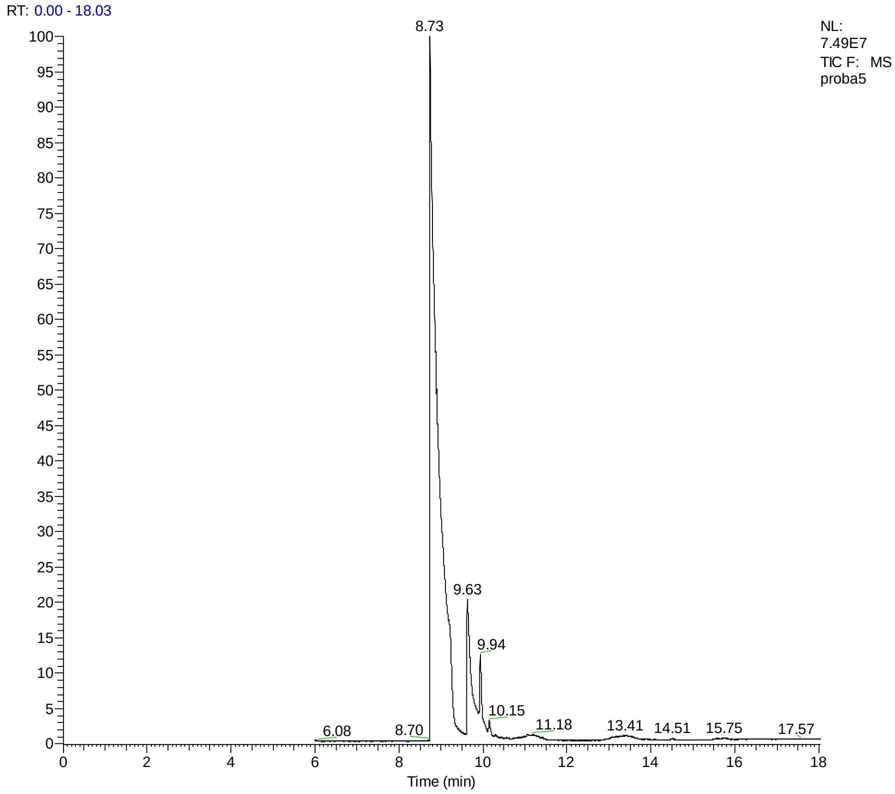

Figure S7. GC-MS spectrum for the products of thermal destruction of polypropylene in a sealed autoclave with H_2O + O_2 at 150 °C.

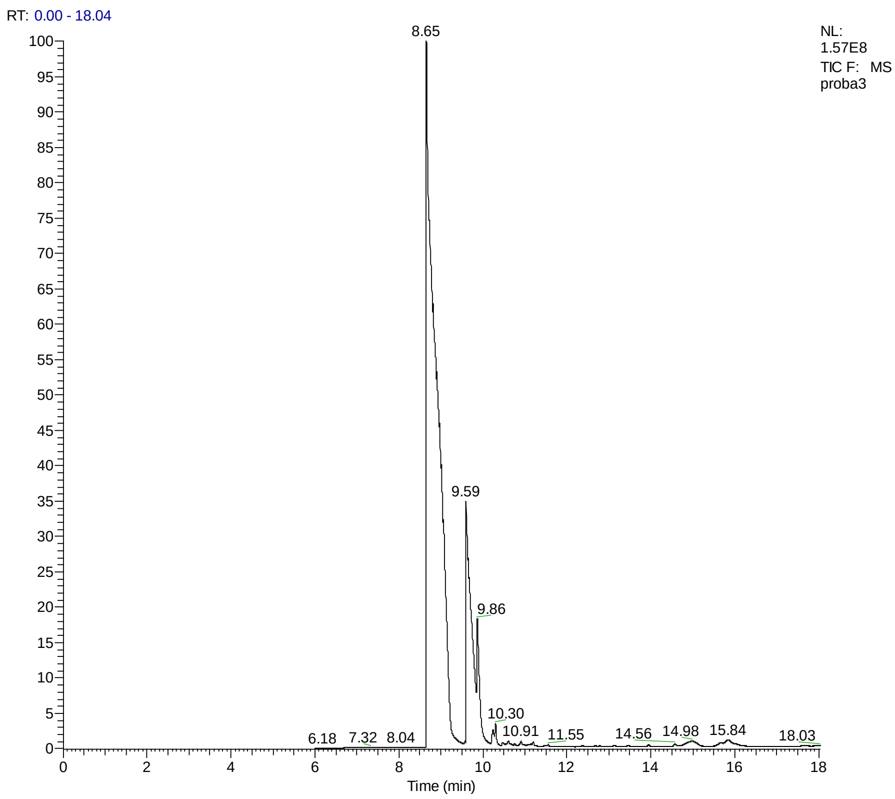

Figure S8. GC-MS spectrum for the products of thermal destruction of polypropylene in a sealed autoclave with $H_2O + O_2 + CO_2$ at 150 °C.

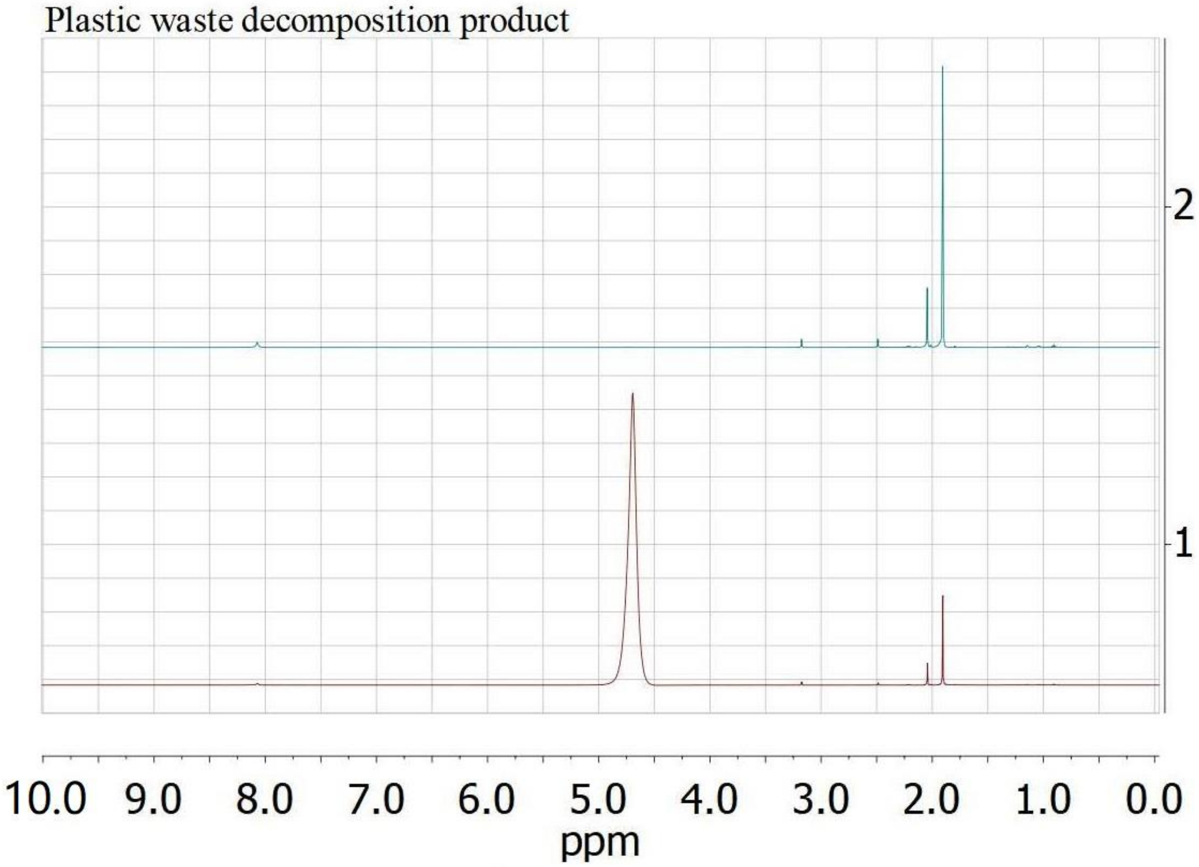

Figure S9. ^{1}H NMR spectra of the product of decomposition of plastic waste. Lower image without water suppression, upper image with water suppression.
